# Asymmetric Coding of Categorical Spatial Relations in Both Language and Vision

**DOI:** 10.3389/fpsyg.2012.00464

**Published:** 2012-11-20

**Authors:** J. C. Roth, S. L. Franconeri

**Affiliations:** ^1^Northwestern UniversityEvanston, IL, USA

**Keywords:** spatial relationships, spatial language, relation perception, binding

## Abstract

Describing certain types of spatial relationships between a pair of objects requires that the objects are assigned different “roles” in the relation, e.g., “A is above B” is different than “B is above A.” This asymmetric representation places one object in the “target” or “figure” role and the other in the “reference” or “ground” role. Here we provide evidence that this asymmetry may be present not just in spatial language, but also in perceptual representations. More specifically, we describe a model of visual spatial relationship judgment where the designation of the target object within such a spatial relationship is guided by the location of the “spotlight” of attention. To demonstrate the existence of this perceptual asymmetry, we cued attention to one object within a pair by briefly previewing it, and showed that participants were faster to verify the depicted relation when that object was the linguistic target. Experiment 1 demonstrated this effect for left-right relations, and Experiment 2 for above-below relations. These results join several other types of demonstrations in suggesting that perceptual representations of some spatial relations may be asymmetrically coded, and further suggest that the location of selective attention may serve as the mechanism that guides this asymmetry.

## Introduction

Throughout cognition, absolute values are less important than relative values. At the earliest levels of perception, our visual system translates local luminance into contrast (Peli, 1990). At the highest levels of cognition, we make decisions about values (e.g., whether a particular gas station’s prices are “cheap”) based on other values serving as a baseline (even when those baseline values are irrelevant; Tversky and Kahneman, 1974). Here we explore an intermediate case – our perceptual system’s representation of the relative spatial positions of objects, e.g., “A is above B.”

The class of relations that we address is the categorical spatial relation. *Categorical* denotes relations where exact metric information is less relevant than the abstracted relational prototypes that objects might fit, such as “left of,” or “above.” For example, a stapler can still be to the left of the keyboard, whether it is 2′′ or 2 feet away (Kosslyn, 1987; Chabris and Kosslyn, 1998). Ratings for how well a pair of objects match a given relational category are subject to their fit within a rough prototype of ideal spatial arrangements, e.g., within an ideal “above” relation, two objects are vertically but not horizontally offset (Hayward and Tarr, 1995; Logan and Sadler, 1996; Regier and Carlson, 2001; Carlson and Logan, 2005).

This class of relations logically requires that objects within the pair are assigned different “roles” in the relation, such that “A is above B” is different than “B is above A” (Miller and Johnson-Laird, 1976). This *asymmetry* property can be expressed within spatial language by the assignment of one object as the “target” or “figure,” and the other as the “reference” or “ground” (e.g., “the target is to the left of the reference”). There are several properties of objects that can guide the assignment of target and reference status (Carlson-Radvansky and Radvansky, 1996; Taylor and Tversky, 1996). As an example, small and movable objects tend to be chosen as targets, in reference to large immobile objects (e.g., Clark and Chase, 1974). It sounds natural to say that “The bike is to the left of the building,” but odd to say that “The building is to the right of the bike” (Talmy, 1983)^1^.

Here we argue that perceptual representations of categorical spatial relations share this property of asymmetry. We first describe an account where visual spatial relations are extracted by monitoring the direction of shifts of the attentional “spotlight” over time. We then suggest that the current location of the attentional spotlight marks one object within a relation being “special,” and this marker may be similar to the asymmetric representation of one object as the “target” within spatial language. To test this possibility, we manipulate attention by cueing one object within a pair. We find that people are faster to verify the relation when this cued object is the “target” within a verbal description, consistent with the idea that the attentional spotlight plays a role in creating a similar asymmetry in the perceptual representation.

### The attentional “spotlight”: A potential mechanism for marking the asymmetry of a relation

We briefly describe a model of visual spatial relationship judgment where the designation of the target object within such a spatial relationship is guided by the location of the “spotlight” of attention (Franconeri et al., 2012). A primary component of a relation between two objects would be networks that represent single objects within the ventral visual stream. This stream is hierarchically organized, such that at lower levels of the stream, networks process incoming visual information in relatively simple ways (e.g., processing local orientation or brightness), while at higher levels, the processing becomes progressively more complex (e.g., shape, curvature; see Grill-Spector and Malach, 2004 for review). At the most complex levels these networks do allow recognition of objects in a way that might be used to encode spatial relations, such as networks that respond to spatial arrangements of facial features, the orientation of a hand, or the presence of a dark blob above a light blob (Tanaka, 2003). However, these representations would not suffice for flexible recognition of relations without such existing representations of a particular pair of objects in a particular arrangement.

Importantly, the ventral stream does not always precisely represent *where* objects are in the visual field. Earlier levels of this stream do focus on local areas of the visual field, and therefore represent location precisely. But later levels represent information from progressively broader areas of the visual field, as large as entire visual hemifields (Desimone and Ungerleider, 1989). Thus, we may know that a cup is present, but we may not know precisely where it is. A proposed solution to this problem is to relatively isolate processing to specific locations in the visual field, so that any features or objects present must be confined to that location in the visual field, amplifying signals from that location while relatively inhibiting signals from other areas (Treisman and Gelade, 1980). Thus, localizing a given object may require that we selectively process its location with the “spotlight” of attention. Evidence for this idea comes from studies where participants are prohibited from focusing their spotlight, resulting in localization errors (Treisman and Schmidt, 1982). In addition, recent studies using an electrophysiological technique that tracks this spotlight have shown that merely identifying objects does not necessarily require selectively processing its location, but localizing even the simplest object does appear to require that we select its location (Luck and Ford, 1998; Hyun et al., 2009). This selection process appears to be controlled by parietal structures in the dorsal visual stream, which is argued to contain a spatiotopic map of the visual field that represents the location(s) selected by the attentional spotlight (Gottlieb, 2007; Serences and Yantis, 2007).

Thus, the ventral stream can represent what objects are present in the visual field, but localizing any individual object appears to require selection of an object’s location. If so, then how might we compare the relative spatial relationship between *two* objects? Intuitively, we feel as if the relation is revealed when we spread our spotlight of attention across both objects at once. In contrast, the evidence above suggests that we must select objects one at a time in order to localize them (as well as to surmount other processing constraints related to object recognition, see Franconeri et al., 2012). We have recently argued for this latter possibility, where spatial relationships are judged with a process that isolates at least one of the objects with selective attention (Franconeri et al., 2012). For example, imagine judging the left/right relation between a red and a green ball. Attending to both objects initially, the ventral stream could represent the fact that a red and a green ball were present in the visual field, and even that they were horizontally arranged (because a blurred version of the objects would contain a horizontal stripe). But this representation does not contain explicit information about the relation between these objects.

**Figure 1:**
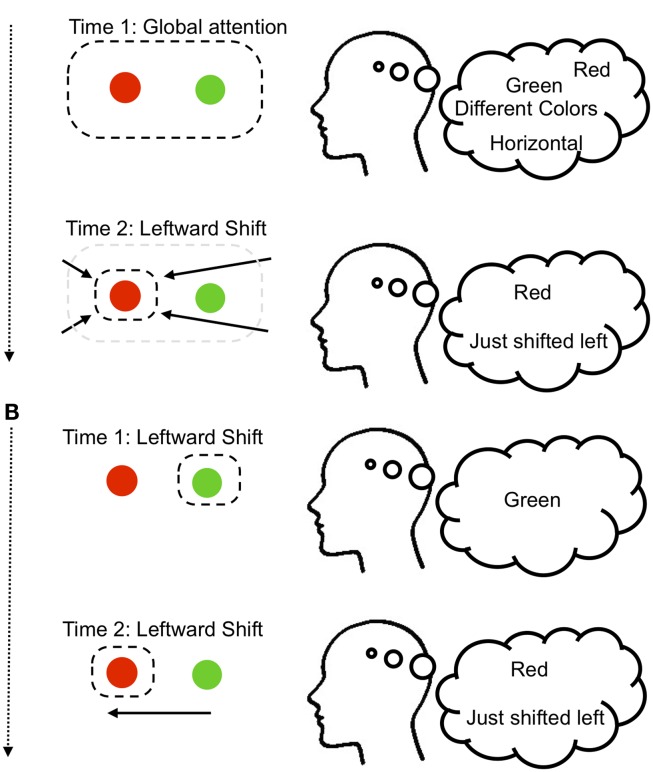
**Two variants of the visual spatial relationship judgment model from Franconeri et al. (2012)**. **(A)** When first encountering a pair of objects, we might select both in a global fashion, resulting in activation of those object identities in the ventral visual stream, perhaps along with other information such as the fact that they differ, or are horizontally arranged. Critically, within this global attentional state we do not know the relative positions of each object. Shifting the spotlight of attentional selection to the left would allow the conclusion that the red object was on the left of the arrangement. **(B)** A second way to encode relations would be to isolate one object (e.g., green), and then shift attention to the other object (e.g., red), recording the direction of the shift (e.g., left).

To recover an explicit representation of the relation, we proposed that the perceptual system might encode the spatial relation by shifting the spotlight of selection toward one of these objects (e.g., the red ball), and encoding the *direction* that the spotlight moved (e.g., to the left; see Figure 1)^2^. Thus, the relations between the objects are encoded first as (red exists, green exists, horizontal arrangement), and then after the attention shift as (red exists + just shifted left). It is also possible that only one of the objects is selectively attended, such that the spotlight starts at, e.g., the green object, producing (green exists), and shifts to produce (red exists + just shifted left). In support of this idea that attention shifts are needed to perceive spatial relations between objects, we used an electrophysiological attention tracking technique to show that during such simple relational judgments, participants do shift their attention in systematic ways toward one of the objects (Franconeri et al., 2012; Xu and Franconeri, 2012).

The attention shift mechanism is not the only possible mechanism that the visual system might employ for judging spatial relationships among objects (see Franconeri et al., 2012, for review; and see Hummel and Biederman, 1992 for an alternative account). But it is a relatively simple and parsimonious solution that makes testable predictions. According to this account, the “visual” representation first contains information about what objects are present and how they are arranged (e.g., horizontally vs. vertically), and then at a different time point this visual representation contains the information that the red object is on the left of whatever region of the visual field was previously attended. Therefore, the representation and understanding of more complex relations (e.g., knowing what the most recent object was left *of*, or understanding relations among even greater numbers of objects) would require broader cognitive systems to guide the selection sequence and store the results of that sequence.

In summary, this model predicts that the location of the spotlight of attention marks one object within a relation as being “special,” and this mark may be similar to asymmetric representation of one object as the “target” within spatial language.

### Linking linguistic and perceptual representations of spatial relations

One source of support for the idea that both linguistic and perceptual representations are asymmetric comes from demonstrations of compatibility effects between the two representation types. For example, Clark and Chase (1972) used “sentence-picture verification” tasks where they asked participants to verify whether statements such as “star is above plus” or “plus is below star” were true of an image (see also Carpenter and Just, 1975; Just and Carpenter, 1976). In a critical experiment, when participants were first shown the image, subsequent verification of statements involving the word “above” were faster than those involving the word “below.” This suggested that the “above” framing, which marked the top object as special, was more consistent with the visual encoding of the picture, implying that the picture’s encoding represented the top object as special. In support of this idea, when participants were asked to focus on the top object in the initial image, this effect remained, but when asked to focus on the bottom object, the effect partially reversed, suggesting that the asymmetry within the visual representation could be changed, and that this change was somehow related to attention.

The sentence-picture verification task offers the advantage that it tests for compatibility between linguistic and perceptual representations. Other tasks can show influences of one representation on the other, though it is not always as clear whether those influences reflect biases as opposed to mandatory interactions. For example, some studies show that linguistic representations can influence perceptual processes as indexed by eye movements. In a visual search task (e.g., finding a red vertical target among red horizontal and green vertical distractors), patterns of response time data suggested that participants were able to make use of fragments of a description of a search target (“Is there a red vertical?”) such that hearing only (“Is there a red…”) allowed them to isolate their search to those objects. This suggests a “fluid interaction” where language could guide attentional allocation (Spivey et al., 2001). In another experiment, preparing to produce different descriptions of a scene affected the ways that the eyes move across that scene (Papafragou et al., 2008). Yet another set of tasks showed that when observers were about to describe an object in a scene, they looked to the object’s position before naming it (Altmann and Kamide, 1999).

Other studies show that perceptual manipulations can affect the way that scenes are described. One study showed a series of fish swimming toward each other, with one always eating the other. If the predator fish (e.g., the red fish) were cued with an arrow, observers were more likely to describe the scene actively (e.g., “The red fish ate the green fish”), whereas if the prey fish (e.g., the green fish) were cued with an arrow, the description was more likely passive (e.g., “The green fish was eaten by the red fish”; Tomlin, 1997). Similarly, another study showed that subtler attentional cues added just before the appearance of a scene could influence descriptions of that scene (Gleitman et al., 2007). In a scene containing a man and a dog, cueing the future location of a dog was more likely to produce descriptions such as “The dog chases the man,” while cueing the future location of the man was more likely to produce “The man flees the dog^3^.”

While these paradigms and results support important conclusions about the strength and timecourse of interactions between language and perception, we used a sentence-picture verification task because it is uniquely suited for seeking compatibility between the representations underlying the comprehension of the picture and the sentence. Also, in contrast with other studies that use several or even dozens of objects within the depicted scenes (e.g., Altmann and Kamide, 1999; Spivey et al., 2001), we used scenes containing only two objects, which is well within any estimate of the processing or memory capacity of the visual system (e.g., Luck and Vogel, 1997; Franconeri et al., 2007). Thus, any effects of attention within such simple scenes should be all the more surprising.

## Experiments

We suggest that visual representations *per se* can be asymmetric, and that the mechanism for marking an object as special is the exclusive attentional selection of its location. We test this idea directly by using attentional cueing manipulations that drag the spotlight toward one of the objects. Because these manipulations are extremely rapid and subtle, and should not create strong demand characteristics that may be present in previous studies (e.g., Tomlin, 1997; see Gleitman et al., 2007, for discussion), they should primarily affect visual representations. Before participants saw the objects, they were given a question to answer about the relation between the objects. For example, if we asked, “Is red on the left of green?” then responses to that question might be faster if the red object appears before the green object, in the same ordering as the question. This question was presented several seconds before a series of trials, so that participants were matching displays to a memory representation of the question, and were not reading it online during the trials.

We found that participants were faster to verify the depicted relation when the cued object was the linguistic target, suggesting that the cueing manipulation affected the format of the visual representation of the relation. Experiment 1 demonstrated this effect for left-right relations, and Experiment 2 for above-below relations. These experiments were similar in spirit to past work on sentence-picture verification suggesting asymmetries in perceptual representations of simple visual relations (e.g., Clark and Chase, 1972), except that we more explicitly tested the role of the location of attention in establishing this asymmetry.

### Experiment 1: Left/Right relations, with one object appearing before the other

We gave participants a statement to verify, followed by eight displays containing red and green objects in both spatial arrangements. To ensure that participants extract *relations* between colors from the display, and not just positions of single objects, we varied the absolute spatial location of both objects in a way that the position of the first object gives no information about the relation between the objects. We manipulated attention by displaying one of the objects briefly before the other (Franconeri et al., 2005).

Questions were shown at the start of each eight-trial block, and were of two possible forms. The first was similar to the ones used by Clark and Chase (1972) (e.g., “Is red left of green?”). For the first question type we focused our analysis on the predicted target/reference compatibility effect, where response times should be faster when the target object (red) is cued, relative to when the reference object (green) is cued. Note that there are other ways in which compatibility effects could arise – we could look for effects of whether the cued object was the object specified by the direction term (e.g., left), an object of a particular color regardless of the question asked, or an object on a particular relative spatial location (e.g., left or top) regardless of the question asked (Tversky et al., 1991; Maas and Russo, 2003; Jahn et al., 2007). We did not have strong *a priori* predictions for these other potential types of compatibility, and there were no robust effects among them. While we focus on the target/reference effects here, analyses and graphs for these other types of compatibility effects are in the Appendix. The second type of question was of the form, “Which color is left?” Here we sought a spatial compatibility effect, but were not sure of its direction – one might expect that precueing the left object would lead to better performance, but then again precueing the right object would lead to “leftward motion” of the objects when the left object appeared second. We found only weak evidence for the former possibility, and we also note additional problems with the interpretation of this effect in the “General Discussion” section. We therefore focus instead on the target/reference effects from the first question type.

Manipulations of attention with transient cues can be sensitive to timing. As pilot data for future studies, we included several levels of asynchrony between the precue display and the full display containing both objects (33–233 ms). However, because there was insufficient power to confidently distinguish among these levels and their varied interactions with different types of compatibility effects, we collapse across these timing differences in the present description, but provide the analyses and graphs in the Appendix.

#### Methods

##### Participants

Thirty-one undergraduate students at Northwestern University participated in the 25-min session in exchange for course credit.

##### Apparatus and stimuli

**Figure 2:**
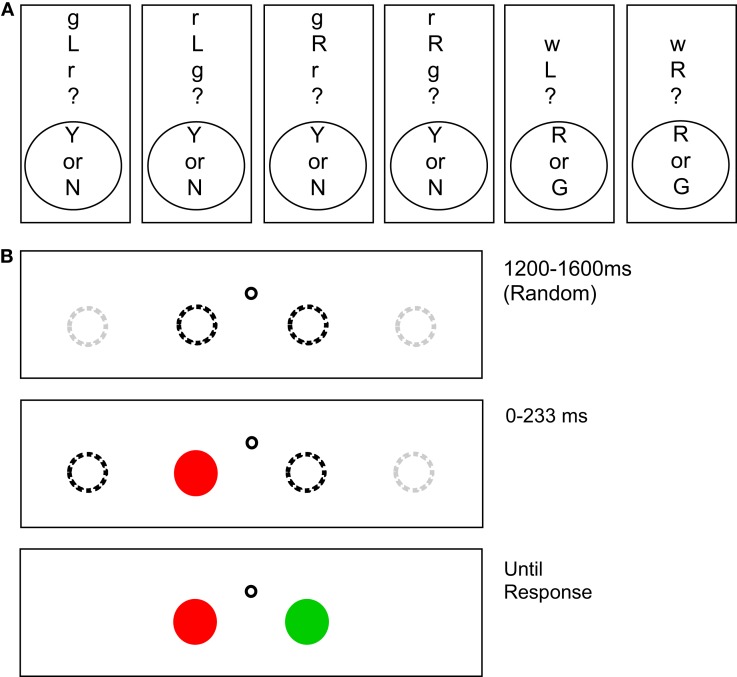
**(A)** Potential instruction displays for Experiment 1. **(B)** Illustration of a potential trial sequence. After a fixation point, one of the objects in the relation appears in either the second or third positions (dotted black lines) of four possible positions (dotted black and gray lines). Because the other object could appear on either side, this display gave no information about the relation between the objects. After a delay (0–233 ms), the second object appears and the participant could give their response.

The experiment was controlled by a Dell Precision M65 laptop computer running SR-Research Experiment Builder. Although head position was not restrained, the display subtended 32.6°× 24.4 at an approximate viewing distance of 56 cm, with a 1024 × 768 pixel resolution, 33.6 pixels per degree. A display with one of six questions was shown, followed by eight trials. Figure 2 depicts examples of questions and test displays. Questions were presented vertically as symbolic letter abbreviations to reduce directional biasing from reading order. The display background was gray (14.2 cd/m^2^). Each trial consisted of the display of two circular targets, each at one of four locations spaced equally in the horizontal direction on the display. A black (1.1 cd/m^2^) circular fixation point with a diameter of 11 pixels was present between the two innermost targets at a distance of 50 pixels to the left or right and 36 pixels above the targets. Each target was 33 pixels in diameter. One circle was always red (19 cd/m^2^) and the other circle was always green (24 cd/m^2^), values that are approximately perceptually equiluminant (see Franconeri et al., 2012).

##### Procedure

There were 288 experimental trials in blocks of eight trials with each trial repeated twice for a total of 576 trials per subject. At the beginning of each block of eight trials, participants were either instructed to indicate whether the relation was correct or incorrect, or which target color was on the specified side. These eight trials consisted of the combination of two starting locations, two starting object color possibilities, times two possible locations for the second object. The order within and among these eight-trial blocks was randomized. Participants were instructed to maintain strict fixation through each trial, even if it this hurt their performance. At the beginning of each trial, a blank display was presented for 800 ms, followed by a fixation point presented for 1200–1600 ms. To minimize timing effects related to pre-trial alerting, participants were then alerted by an auditory signal 200 ms prior to the appearance of the first object. One object was displayed, and then the second object appeared either 0 (simultaneously), 33, 83, 133, 183, or 233 ms later. Instructions were in two different forms (see Figure 2). The first was, “is X (Direction) of Y?” where X and Y were “r” for red and “g” for green and (Direction) was “L” for left and “R” for right. Questions were displayed as “X(Direction)Y?” Participants responded with yes or no using the Y and N keys on the keyboard. The second form of instructions was, “Which is (Direction)?” In this question type (Direction) was “L” for left and “R” for right. Questions were displayed as “w(Direction)?” For these trial types, participants responded with red or green using the R and F keys on the keyboard. The F key was used instead of the G key for green so that the vertical arrangement of the keys on the keyboard could reduce potential directional biasing. The F key was labeled with a letter G to avoid confusion.

#### Results and discussion

**Figure 3:**
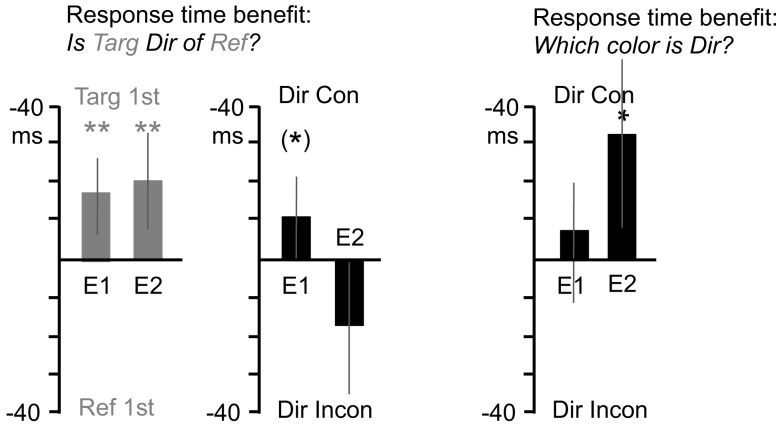
**Response time benefits for Experiments 1 and 2**. The first graph (gray bars), depicts response times advantages for the “Is Target Direction of Reference?” question type. Values toward graph top indicate faster responses for trials where the “target” object appeared first, and values toward graph bottom indicate faster responses for trials where the “reference” object appeared first. In the second graph (black bars), values toward graph top indicate faster responses for trials where object consistent with the directional term (e.g., the left object for “left” questions) appeared first, and values toward graph bottom indicate faster responses for trials of the opposite case. The third graph (black bars) depicts response time advantages for the “Which Color is Direction?” question type, using the same “direction consistency” analysis as the second graph. Asterisks indicate significant effects, an asterisk in parentheses indicates a marginal effect.

Three subjects were omitted from the analysis due to a failure to complete the experiment. One subject was omitted due to particularly low accuracy (85%), and one was omitted due to an average response time more than 2 SDs above the mean. Twenty-six subjects remained in the analysis. Accuracy rates were 96% (SD = 1.94%) and average response time was 755 ms (SD = 129 ms). Trials with incorrect responses or responses of over 1500 ms were removed from the analysis. Figure 3 depicts the main effects within the data collapsed over the timing manipulations. Note that the Figure does not depict “baseline” RTs, only differences in RT between different conditions. Analysis of variance below will include baseline RT as a factor, and significant changes in RT across timings are described in the Appendix.

##### Is (target) (direction) of (reference)

The left side of Figure 3 shows analyses of questions of the form “Is (target) (direction) of (reference)?” Showing the target object before the reference object led to faster response times. For the *target/reference* analysis, response times were submitted to a 2 × 6 repeated measures analysis of variance, with object appearance order (target first, reference first) and timing (0, 33, 83, 133, 183, 233 ms) as variables. Responses were faster when the question’s target object appeared first (*M* = −15.5 ms) supported by a significant main effect of object appearance order *F*(1, 25) = 9.2, *p* = 0.006. For the direction term consistency analysis, response times were submitted to a 2 × 6 repeated measures analysis of variance, with direction term consistency (direction term consistent with object that appears first, e.g., “left” when left object appears first, or inconsistent) and timing as variables. There was a trend for direction consistency to improve response times (*M* = −10.2 ms), *F*(1, 25) = 3.5, *p* = 0.07. See Appendix for additional analyses.

##### Which is (Direction)

The right side of Figure 3 shows analyses of questions of the form “Which is (Direction)?” There was no main effect of *direction term consistency* (whether the first object appeared on the side named by the directional term). A 2 × 6 repeated measures analysis of variance, with direction term consistency and timing as variables, revealed no main effect of direction consistency on response times (*M* = −8.8 ms), *F*(1, 25) = 0.8, *p* = 0.4. See Appendix for additional analyses.

### Experiment 2: Above/Below relations, with one object appearing before the other

Experiment 2 was identical to Experiment 1, except that objects were arranged vertically instead of horizontally. The questions shown before each block of eight trials were now depicted horizontally so that reading order would be orthogonal to the dimension of the judged relation.

#### Methods

##### Participants

Fourteen undergraduate students at Northwestern University participated in the 25-min session in exchange for course credit.

##### Apparatus and stimuli

Stimuli were identical to those in Experiment 1 except that the objects were aligned vertically instead of horizontally, horizontally centered on the display, with the fixation point in between the middle two objects. Because the vertically-oriented question displays from the previous experiment would now have a confounded reading order, the present displays used horizontally oriented questions, now written out in standard English.

##### Procedure

The procedure was similar to that of Experiment 1 with the following exceptions. The trials were blocked by timing between the appearance of the two objects, and the order of these blocks was randomized. For questions of the form, “is X (Direction) of Y?” X and Y were “red” or “green” and (Direction) was “Above” and “Below.” Questions were displayed as “X (Direction) Y?” Participants responded to these questions using Y (Yes) and U (No) keys on the keyboard. The U key was labeled with the letter “N,” and was used instead of the N key so that the horizontal arrangement of the keys on the keyboard could further reduce directional biasing. For questions of the form, “which is (Direction)?” (Direction) was “Above” and “Below.” Questions were displayed as “which is (Direction)?” For these trial types, participants responded with red or green using the R and T keys on the keyboard. The T key was used instead of the G key for green so that the horizontal arrangement of the keys on the keyboard could further reduce directional biasing. The T key was labeled with a letter “G” to avoid confusion.

#### Results and discussion

Accuracy rates were 95% (SD = 2.54%) and average response time was 767 ms (SD = 110 ms). Trials with incorrect responses or responses of over 1500 ms were removed from the analysis. Figure 3 depicts the various ways that data were collapsed for analysis.

##### Is (target) (direction) of (reference)

Figure 3 shows analyses of questions of the form “Is (target) (direction) of (reference)?” identical to those for Experiment 1. Again, displaying the target object before the reference object led to faster response times. Response times for the *target/reference* analysis were submitted to a 2 × 6 repeated measures analysis of variance, with object appearance order and timing as variables. Responses were faster when the question’s target object appeared first (*M* = −19.3 ms), supported by a significant main effect of object appearance order *F*(1, 13) = 9.3, *p* = 0.009. For the *direction term consistency* analysis, there was a marginal consistency advantage. Response times were submitted to a 2 × 6 repeated measures analysis of variance, with direction term consistency and timing as variables. The main effect of direction consistency was marginally significant (*M* = 18.3 ms), *F*(1, 13) = 4.0, *p* = 0.07. See Appendix for additional analyses.

##### Which is (Direction)

The right sides of Figure 3 shows analyses of questions of the form “Which is (Direction)?” and here there was a main effect of direction consistency such that responses were faster when the first object appeared on the side named by the directional term. Response times for the *direction term consistency* analysis were submitted to a 2 × 6 repeated measures analysis of variance, with direction term consistency and timing as variables. There was a main effect of direction consistency on response times (*M* = −31.4 ms), *F*(1, 12) = 5.4, *p* = 0.04, reflecting an advantage when objects appeared in a direction consistent with the term used in the question. See Appendix for additional analyses.

## General Discussion

We tested whether the position of the attentional spotlight affects visual representations of relations by determining the direction of asymmetry within that relation. Experiment 1 tested left/right relations, while Experiment 2 tested above/below relations. We manipulated attention by precueing one object within the pair, and this precue affected compatibility with the linguistic framing of the question that participants were asked to verify. The most robust example was the type of compatibility for which we had a strong *a priori* prediction – target/reference designations for questions of the form, “Is (target) (direction) of (reference)?” In both experiments, participant responses were faster when the “target” object appeared before the “reference” object, an order that follows the ordering within the question.

For these questions, there were no robust effects suggesting response time advantages when the direction term in the question was consistent with appearance order. For questions of the form “Which is (Direction)?” both experiments show some response time advantages when the first object appears on the side named by the direction term (e.g., for “Which is left,” responses are faster when the left object appears first). This effect was weak in Experiment 1 (specific to one timing value, see Appendix), and was a main effect for Experiment 2. However, the direction consistency benefits from these simpler questions are more difficult to interpret. If the results had shown the opposite effect, such that response times were faster when the *second* object appeared on the side named by the directional term, it could have indicated an advantage for trials where the attentional “spotlight” traveled in that direction. For example, when asking, “Which is left,” some versions of the attentional shift account would predict better performance after a *leftward* shift, which should simultaneously produce the representation of the shift direction (left) plus the color of the object on that side of the relation. But because the results suggest that the identity of the *first* object matters, we cannot rule out the possibility that the preview of the first object primed the response to that object’s color identity, regardless of any effects of that preview on relational processing. Thus, given the current results we cannot draw any firm conclusions from these directional questions.

In summary, the most diagnostic results stem from the target/reference analysis across the “Is (target) (direction) of (reference)?” questions, which show that previewing the linguistic target object slightly before the reference object (the same temporal ordering as the question) speeds response times for both left/right and above/below judgments. Attentional manipulations do affect the compatibility of visual representations with asymmetric linguistic representations, providing evidence that visual representations of relations may be similarly asymmetric.

Note that following the order prescribed by the sentence reveals an interesting potential property of the perceptual representation: following that order produces the “wrong” relational term within the attention shift model. For example, given an image of (red green) and the question “Is red left of green?” according to our account, following the order of the question would produce (red) and then (rightward + green). For this representation to be compatible with the surface form of the linguistic representation, the visual mechanism would have to “flip” the directional term (changing “right” to “left”). This flip is counterintuitive, but certainly not computationally difficult.

A deeper understanding of how these asymmetries interact will require additional converging evidence for how attention moves within such simple displays of relations, as well as new data using other types of cueing methods (e.g., transient events that occur near or on two existing objects, instead of having one object appear at a different time point). Ongoing work in our laboratory does show that using other measures besides attentional cueing (eyetracking and electrophysiological attention tracking techniques), we find that the eyes and attention are controlled in the same ways as suggested here. That is, when engaged in a sentence-picture matching task, the eyes (and attention) shift toward the relational “target” object (Franconeri et al., 2012). Note that, by themselves, such tracking results could not show a causal effect of attentional allocation in the way that the present studies do.

The idea that visual relation representations are asymmetric is consistent with our account of visual relation processing (Franconeri et al., 2012), which predicts that the visual system provides a serial stream of information about the relations between objects in a scene, one relation at a time. If visual relations are processed in such a serial fashion, why do we feel as if we have a more detailed percept of the relations around us? One possibility is that other visual information about the objects within the relation supports this percept of detail, such as how many are present (Franconeri et al., 2009), the global shape of their arrangement (Sanocki and Sulman, 2009), and statistical information about their identities (Ariely, 2001). Individual relations may be produced “on demand” so quickly that they give the conscious impression that they were already available (Rensink et al., 1997; Noe and O’Regan, 2000). For example, an observer might automatically process both “sides” of an asymmetric relation to know both that red is left *and* green is right, such that the percept of that relation feels symmetric.

Given the present results, how certain could we be that perceptual representations of relations are asymmetric? For example, what if the attentional cueing manipulation affected an intermediate (and asymmetric) representation between perception and language, while perceptual representations are actually symmetric? While we cannot rule out this possibility, we find our conclusion more parsimonious. Given what is known about how the visual system might process spatial relationships, we argue *a priori* that its representation of categorical relations should be asymmetric. Without a specification of the nature of this potential “intermediate” representation, this alternative account seems difficult to falsify. Furthermore, this intermediate representation would also have to be affected by the precueing manipulation, which participants knew to be irrelevant to their task, and which should primarily affect perceptual representations. Another similar critique would be that the perceptual representation is symmetric, but that translating that representation into one that is compatible with language requires that it be somehow reformatted, e.g., to match the serial order of the sentence. If so, then a more conservative version of our claim would be that the subset of perceptual representations that potentially interfaces with language is asymmetric. But this subset would include the vast majority (and perhaps all) *useful* spatial relationship judgments that the mind constructs.

### Must perceptual relation representations be asymmetric?

There may be other types of “relational” perceptual representations that do not involve the same type of asymmetry as suggested here. In particular, there may be visual representations that allow other forms of relational information to be represented in a less explicitly asymmetric way. For example, some models of performance in similar tasks specify underlying perceptual representations that are more “holistic” or “pictorial” than linguistic descriptions given enough encoding time (e.g., Seymour, 1969; Tversky, 1975; Glushko and Cooper, 1978; Reichle et al., 2000; see also similar issues regarding representations underlying mental imagery, e.g., Anderson, 1978; Kosslyn, 1994; Pylyshyn, 2003). One study demonstrated that when a sentence was shown before the picture, and participants had plenty of time to recode the depicted relations into a “pictorial” format, the markedness and sentence complexity effects that underlie the claims of studies such as Clark and Chase (1972) disappear. Instead, these authors argued that participants could match the subsequent image with a pictorial representation in a holistic manner (Glushko and Cooper, 1978).

How do such results relate to the present evidence for asymmetric perceptual representations? We believe that while it might be possible to create a pictorial representation that could be compared against a subsequent picture, it will only be possible if the “relation” has an existing holistic representation in the ventral visual system. For example, in the Glushko and Cooper (1978) study, the type of stimuli used (a small set of close arrangements of squares and triangles) could be recoded into global shapes (e.g., the relation of “triangle above square1; square2 left of square1” would be uniquely identifiable as a global “L” shape). In another study making similar claims, the “relation” of eyes above a mouth in a cartoon face almost certainly has an existing holistic representation in the visual system, which could explain the ability of participants to quickly build “holistic” representations of cartoon faces of different shapes (Tversky, 1969). Such well-learned stimuli contrast with the more arbitrary color pair relations used in studies such as Clark and Chase (1972) as well as the present studies. In those cases, it is less clear how *relations* among objects could be represented holistically or pictorially.

### To what extent does perception lean on language?

How could a serial attentional process be controlled, and how could the intermediate and final results of this routine be stored? One primary system underlying the solution to each of these problems may be language itself, or a similar representation that could store more complex or hierarchical relations. More complex relations among multiple objects would require language, or a language-like representation (Cavanagh, 2004), to guide the spotlight of selection across objects, or groups of objects, in the proper order to produce the needed conclusion (e.g., the group containing the red object left of the green object is under the blue bar), and to store the results of that sequence (see Landau et al., 2010, for a review of similar arguments). This possibility is consistent with the close ties between the movements of the eyes and attention within scenes and the comprehension and production of linguistic descriptions of those scenes (e.g., Altmann and Kamide, 1999, 2007; Gleitman et al., 2007).

Our account of visual relation processing may actually *require* a system such as language to deal with all but the most primitive relations. Two recent studies suggest that language may indeed play a role in creating representations of visually presented spatial relations. In one study, hearing spatial language helped children encode the spatial relations among a set of three objects, allowing them to pick out objects in the same relational role (as opposed to the same identity) in a second set of objects (Loewenstein and Gentner, 2005). In another, when children were given objects that were red on one side and green on the other, performance in a later matching task showed good memory for the shape of the object, but poor performance for the left-right spatial relation between the colors. Adding spatial language during the encoding display improved their performance when that language included information about the color and direction of one of the objects (e.g., “the red is on the left”) (Dessalegn and Landau, 2008). Language may guide attention through the relation in a way that creates a more robust short-term representation of that relation, as opposed to a less optimal strategy of attempting to attend to multiple objects at once. In addition to language serving as a guide for attention, it may also serve to ensure that the sequence of guidance is the same across encoding and test periods, so that the children do not attempt to compare an encoded relation of “the red is on the left of green” with a *different* representation of “the green is on the right of red.”

## Conflict of Interest Statement

The authors declare that the research was conducted in the absence of any commercial or financial relationships that could be construed as a potential conflict of interest.

## Figures and Tables

**Figure A1:**
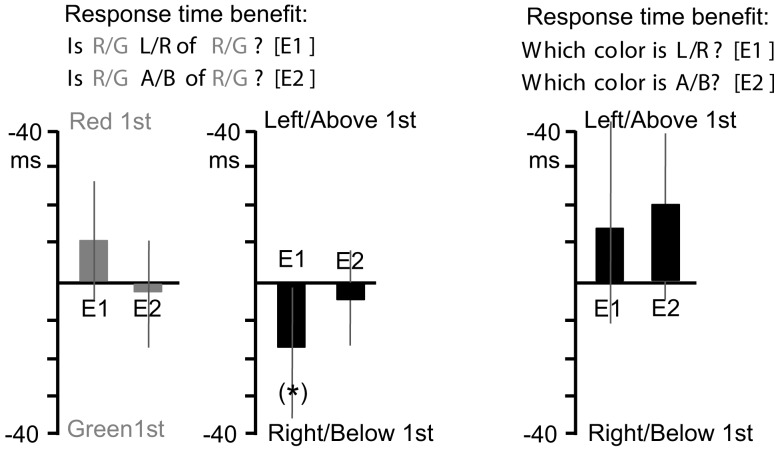
**Response time benefits for Experiments 1 and 2**. The first graph (grey bars), depicts response times advantages for the “Is Target Direction of Reference?” question type. Values toward graph top indicate faster responses for trials where the Red object appeared first, and values toward graph bottom indicate faster responses for trials where the Green object appeared first. In the second graph (black bars), values toward graph top indicate faster responses for trials where Left (E1) or Above (E2) object appeared first, and values toward graph bottom indicate faster responses for trials of the opposite cases. The third graph depicts equivalent results for the “Which Color is Direction?” question type. *Indicates a single marginal effect (which would not survive a multiple comparisons correction).

**Figure A2:**
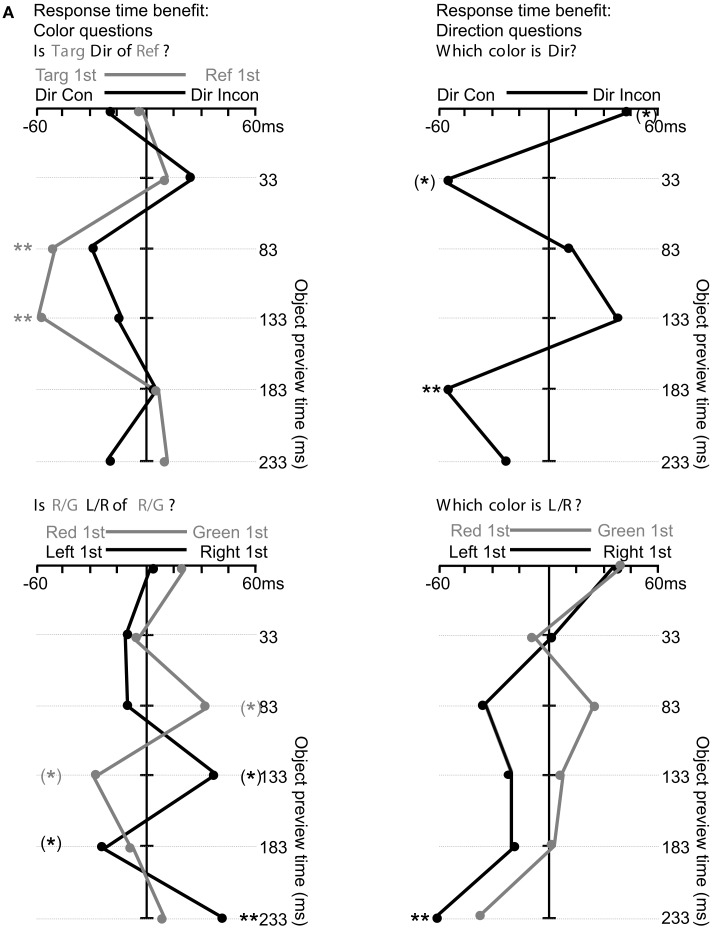

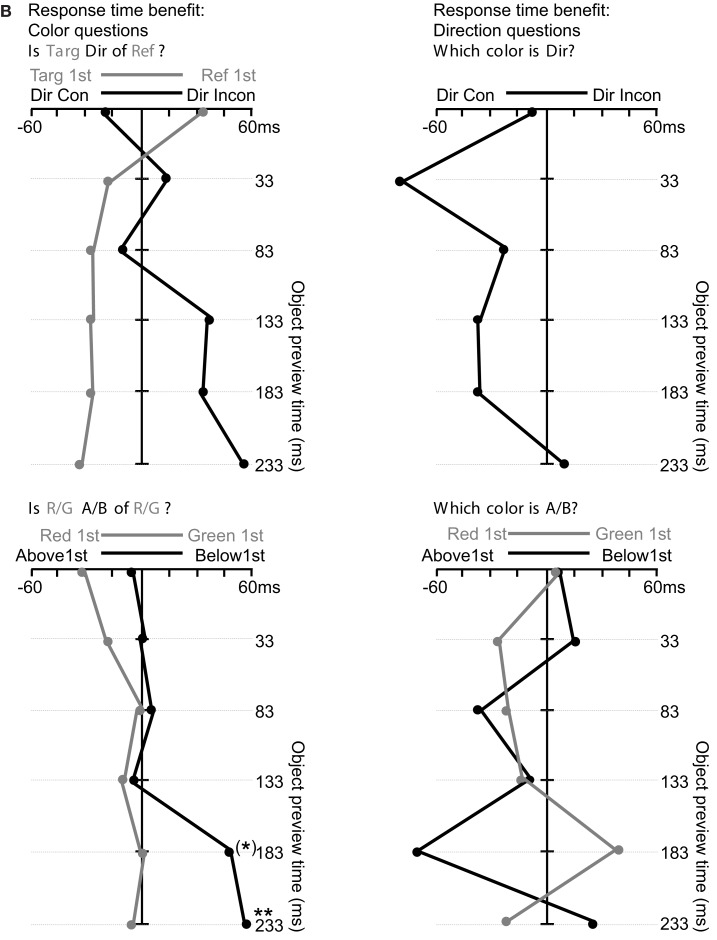
**(A)** Response time results for Experiment 1 (Left/Right relations). **Indicates an effect passing Bonferroni correction *p* < 0.008 (*), indicates 0.008 < *p* < 0.05. **(B)** Response time results for Experiment 2 (above/below relations). **Indicates an effect passing Bonferroni correction *p* < 0.008 (*), indicates 0.008 < *p* < 0.05.

## Footnotes

^1^ Though we focus on this particular type of spatial language, we also note that such asymmetries are not constrained to this class of spatial language, or even spatial language in general - they can apply to a large set of linguistic predicates, depending on syntactic, semantic, and contextual factors (see Gleitman et al., 1996).

^2^ We assume a retinotopic reference system, which is adequate for performing most relational judgments in a glance. For discussion of other types of relational judgments where a retinotopic frame would not seem ideal (e.g., how one might compute a depth relation), see Franconeri et al., 2012.

^3^ But see Griffin and Bock (2000) for an argument for weaker interactions between early stages of scene perception and the construction of linguistic descriptions of scenes, and Gleitman et al. (2007) for detailed discussion of the differing conclusions.

## Appendix

### Alternative compatibility analyses and precue timing analyses

#### Timing analyses: methods

Manipulations of attention with transient cues can be sensitive to timing. The cues take some time to have an effect (typically at least 150 ms), and fade quickly (after around 300 ms), and can even result in an inhibitory effect after even more time has passed (Nakayama and Mackeben, 1989; Klein, 2000). Therefore, in addition to manipulating which object appeared first (was cued), we also systematically manipulated the timing of this event. We added this manipulation for exploratory purposes, and had no *a priori* expectations about how they might affect the patterns of response times. While much is known about how these timing manipulations affect processes such as object identification, it is difficult to predict how they might affect more complex processing of spatial relationships, and the integration of those representations with linguistic descriptions. A detailed look at interactions involving timing will lead to lower power because our design produces seven ways of examining potential asymmetries that might be present in the visual representation (four for the “Is red left of green?” style questions: target vs. reference, directional term consistency, absolute color preference, absolute direction preference; three for the “Which color is left?” style questions: same as above but without target vs. reference). These seven asymmetry types times the six timings produce 42 condition averages for each of the two experiments, and therefore a high likelihood of type I (false positive) errors. We therefore include this manipulation for exploratory purposes for future work, and focus primarily on the main effect of which object is cued instead of the timing of that cue. All timing interactions are reported with a conservative Bonferroni correction.

### Experiment 1: Full data analysis

Three subjects were omitted from the analysis due to a failure to complete the experiment. One subject was omitted due to particularly low accuracy (85%), and one was omitted due to an average response time more than 2 standard deviations above the mean. Twenty-six subjects remained in the analysis. Accuracy rates were 96% (SD = 1.94%) and average response time was 755 ms (SD = 129 ms). Trials with incorrect responses or responses of over 1500 ms were removed from the analysis. Figure 3 depicts the main effects within the data collapsed over the timing manipulations. Figure A1 depicts main effects for analyses discussed below but not in the manuscript. Note that these figures do not depict “baseline” RTs, only differences in RT between different conditions. ANOVA analysis below will include baseline RT as a factor, and significant changes in RT across timings are described in the text. In Figure A2A, the average left/right position of a line collapsed across the y-axis of timing reflects the main effect of the factors described on the *x*-axis. The shape of the line reflects the interaction between that factor and timing.

#### Is (target) (direction) of (reference)

The left side of Figure A2A shows analyses of questions of the form “Is (target) (direction) of (reference)?” The upper left graph focuses on the compatibility between the question structure and the appearance order of the objects. The analysis was collapsed according to whether the question’s target or reference object appeared first (the gray lines), and according to whether the direction term queried (left or right) was consistent with the relative position of the object that appeared first (the black lines). Both of these analyses collapse across the other aspect of the question, as well as the absolute left/right order of animation, and the absolute red/green color of the object. Note that these potential cues for selecting one object are orthogonal to each other, and therefore compete. We predicted that for these question types, one of the cues would be likely to dominate the other.

Data for the *target/reference* analysis (gray line in upper left graph of Figure A2A) were submitted to a 2 × 6 repeated measures analysis of variance, with object appearance order (target first, reference first) and timing (0, 33, 83, 133, 183, 233 ms) as variables. Responses were faster when the question’s target object appeared first (*M* = −15.5 ms) supported by a significant main effect of object appearance order *F*(1, 25) = 9.2, *p* = 0.006. There were also differences in RT (not visible in the graph) across timings, revealed by a main effect of timing *F*(5, 125) = 8.4, *p* < 0.001. This main effect of timing is similar for the remaining analyses and will be omitted below. These differences were driven by faster response times for longer animation timings (e.g., 233 ms), in a roughly linear trend. There was also an interaction between object appearance order and timing (visible in the variability of the gray line in the upper left graph of Figure A2A), *F*(5, 125) = 3.4, *p* = 0.007, reflecting a larger advantage when the target appeared first at the 83 ms [*t*(25) = 2.8, *p* = 0.009] and 133 ms [*t*(25) = 3.8, *p* < 0.001] timing, with both values passing the Bonferroni correction. Data for the *direction term consistency* analysis (black line in upper left graph of Figure A2A) were submitted to a 2 × 6 repeated measures analysis of variance, with direction term consistency (direction term consistent with object that appears first, e.g., “left” when left object appears first, or inconsistent) and timing as variables. There was a trend for direction consistency to improve response times (*M* = −10.2 ms), *F*(1, 25) = 3.5, *p* = 0.07. There was no interaction between direction term consistency and timing, *F*(5, 125) = 1.6, *p* = 0.2.

The analyses of the bottom left graph of Figure A2A ignore the structure of the question, and instead focus on which color appeared first within the display (red or green), which would reveal a processing priority for a given color, or which side the first object appeared on, which would reveal a processing priority for a given side of appearance. Both of these analyses collapse across the other aspect of the display. Data for the *color priority* analysis (gray line in lower left graph of Figure A2A) were submitted to a 2 × 6 repeated measures analysis of variance, with color appearance order (red first, green first) and timing as variables. There was no main effect revealing an advantage for a given color across timings (*M* = 2.0 ms), *F*(1, 25) = 0.08, *p* = 0.8. The interaction between object appearance order and timing was significant, *F*(5, 125) = 2.5, *p* = 0.03, reflecting faster RTs for trials where red appeared first at the 133 ms timing [*t*(25) = 2.3, *p* = 0.03] and where green appeared first at the 83 ms timing [*t*(25) = 2.6, *p* = 0.02].

Data for the *direction priority* analysis (black line in lower left graph of Figure A2A) were submitted to a 2 × 6 repeated measures analysis of variance, with direction appearance order (left first, right first) and timing as variables. There was no main effect of direction appearance order (*M* = 5.3 ms), *F*(1, 25) = 0.7, *p* = 0.4. The interaction between object appearance order and timing was significant, *F*(5, 125) = 3.4, *p* = 0.007, reflecting faster RTs for trials where the object on the right appeared first at the 233 ms [*t*(25) = 3.0, *p* = 0.006] and 133 ms [*t*(25) = 2.6, *p* = 0.01] timings, and faster RTs for trials where the object on the left appeared first at the 183 ms timing [*t*(25) = 2.0, *p* = 0.06]. Only the 233 ms timing difference passed the Bonferroni correction.

In summary, for the questions of the form “Is (target) (direction) of (reference)?” showing the target object before the reference object led to faster response times. While we examine differences within appearance order timings with caution, these differences seem to be driven by the 83 and 133 ms timings. When collapsing across these questions, there were no main effects of the color or relative position of the first object. While there were hints of interactions among these factors with timing, none of these effects reached a sufficiently high significance threshold except for an advantage for displays where the right object appeared 233 ms before the left object.

#### Which is (Direction)

The right side of Figure A2A shows analyses of questions of the form “Which is (Direction)?” For the graphs at the upper right of Figure A2A, the analysis is identical to the graph at the upper left of Figure A2A, except that the target/reference analysis (gray line in the upper left graph of Figure A2A) is omitted. For the graph at the lower right of Figure A2A, analysis is identical to the graph at the lower left of Figure A2A.

Data for the *direction term consistency* analysis (black line in upper right graph of Figure A2A) were submitted to a 2 × 6 repeated measures analysis of variance, with direction term consistency and timing as variables. There was no main effect of direction consistency on response times (*M* = −8.8 ms), *F*(1, 25) = 0.8, *p* = 0.4. There were baseline RT differences across timings (not visible in graph), *F*(3.6, 88.8) = 4.5, *p* = 0.004 (Greenhouse Geisser correction). These differences were driven by RTs that were significantly higher than average (*M* = 664 ms) for the 0 ms timing [*t*(25) = 3.5, *p* = 0.002], as well as RTs that were significantly less than the average for the 233 ms timing [*t*(25) = 2.5, *p* = 0.02]. There was also an interaction between direction term consistency and timing, *F*(3.4, 85.3) = 4.0, *p* = 0.008 (Greenhouse Geisser correction), reflecting faster RTs for trials with direction consistency at the 183 ms [*t*(25) = 2.9, *p* = 0.008], 33 ms [*t*(25) = 2.2, *p* = 0.04] and 0 ms [*t*(25) = 2.6, *p* = 0.02] timings. The significance of this last test demonstrates why we examine any condition × timing interactions in an exploratory and speculative fashion. This test suggests a difference between two conditions that we know to be identical, and is therefore a false positive (to be expected given the 42 such potential tests in each experiment). Thus, in the Section “Discussion” section below, we discuss only the interaction found for the 183 ms timing, which was significant according to a more conservative Bonferroni-corrected threshold (*p* = 0.008).

Data for the *color priority* analysis (gray line in lower right graph of Figure A2A) were submitted to a 2 × 6 repeated measures analysis of variance, with color appearance order (red first, green first) and timing as variables. There was no main effect revealing an advantage for a given color across timings (*M* = 3.2 ms), *F*(1, 25) = 0.4, *p* = 0.5. The interaction between object appearance order and timing was not significant, *F*(5, 125) = 0.8, *p* = 0.6. Data for the *direction priority* analysis (black line in lower right graph of Figure A2A) were submitted to a 2 × 6 repeated measures analysis of variance, with direction appearance order (left first, right first) and timing as variables. There was no main effect of direction appearance order (*M* = −18.0 ms), *F*(1, 25) = 2.8, *p* = 0.1. There was a significant interaction between object appearance order and timing, *F*(5, 125) = 3.0, *p* = 0.01, reflecting faster RTs for trials where the object on the left appeared first at the 233 ms timing [*t*(25) = 3.7, *p* = 0.001].

In summary, for the simpler questions of the form “Which is (Direction)?” there was no main effect of direction consistency (whether the first object appeared on the side named by the directional term), but there was a direction consistency × timing interaction driven by a significant consistency advantage at the 183 ms timing. For the analyses that collapse across question, there were no main effects of the color or relative position of the first object, though there was a relative advantage when the left object appeared 233 ms before the right object.

### Experiment 2: Full data analysis

Accuracy rates were 95% (SD = 2.54%) and average response time was 767 ms (SD = 110 ms). Trials with incorrect responses or responses of over 1500 ms were removed from the analysis. Figure 3 depicts the main effects within the data collapsed over the timing manipulations. Figure A1 depicts main effects for analyses discussed below but not in the manuscript.

#### Is (target) (direction) of (reference)

The left side of Figure A2B shows analyses of questions of the form “Is (target) (direction) of (reference)?” identical to those for Experiment 1. Data for the *target/reference* analysis (gray line in upper left graph of Figure A2B) were submitted to a 2 × 6 repeated measures analysis of variance, with object appearance order and timing as variables. Responses were faster when the question’s target object appeared first (*M* = −19.3 ms), supported by a significant main effect of object appearance order *F*(1, 13) = 9.3, *p* = 0.009. There were also differences in RT across timings, revealed by a main effect of timing *F*(5, 65) = 2.9, *p* = 0.02. These differences were driven by RTs that were greater than the average (*M* = 695 ms) for the 0 ms timing [*t*(13) = 3.2, *p* = 0.008], as well as RTs that were less than the average for the 183 ms timing [*t*(13) = 2.2, *p* = 0.05]. There was also an interaction between object appearance order and timing, *F*(5,65) = 2.4, *p* = 0.05, reflecting a lack of target consistency advantage when the target appeared first at the 0 ms timing. Data for the *direction term consistency* analysis (black line in upper left graph of Figure A2B) were submitted to a 2 × 6 repeated measures analysis of variance, with direction term consistency and timing as variables. There was no main effect of direction consistency (*M* = 18.3 ms), *F*(1, 13) = 4.0, *p* = 0.07. There was no interaction between direction term consistency and timing, *F*(5, 65) = 1.9, *p* = 0.1.

Data for the *color priority* analysis (gray line in lower left graph of Figure A2B) were submitted to a 2 × 6 repeated measures analysis of variance, with color appearance order and timing as variables. There was no main effect revealing an advantage for a given color across timings (*M* = −11.4 ms), *F*(1, 13) = 1.7, *p* = 0.2. The interaction between object appearance order and timing was not significant, *F*(5, 65) = 0.5, *p* = 0.8. Data for the *direction priority* analysis (black line in lower left graph of Figure A2B) were submitted to a 2 × 6 repeated measures analysis of variance, with direction appearance order and timing as variables. There was a trend for a main effect of direction appearance order (*M* = 17.4 ms), *F*(1, 13) = 4.4, *p* = 0.06, reflecting faster RTs when the object on the bottom appeared first. The interaction between object appearance order and timing was significant, *F*(5, 65) = 2.7, *p* = 0.03, reflecting faster RTs for trials where the object on the bottom appeared first at the 233 ms [*t*(13) = 6.2, *p* < 0.0001] and 183 ms [*t*(13) = 2.2, *p* = 0.05] timings.

In summary, for the questions of the form “Is (target) (direction) of (reference)?” displaying the target object before the reference object led to faster response times. When collapsing across these questions, there were no main effects of the color of the first object, but there were effects involving the position of this object. Specifically, response times were faster when the bottom object appeared first, and this advantage was driven by the longest timings, most robustly the trials were there was a 233 ms preview of the bottom object.

#### Which is (Direction)

The right side of Figure A2B shows analyses of questions of the form “Which is (Direction)?” Data for the *direction term consistency* analysis (black line in upper right graph of Figure A2B) were submitted to a 2 × 6 repeated measures analysis of variance, with direction term consistency and timing as variables. There was a main effect of direction consistency on response times (*M* = −31.4 ms), *F*(1, 12) = 5.4, *p* = 0.04, reflecting an advantage when objects appeared in a direction consistent with the term used in the question. There were no baseline RT differences across timings, *F*(5, 60) = 0.3, *p* = 0.9. There was no interaction between direction term consistency and timing, *F*(5, 60) = 1.6, *p* = 0.2.

Data for the *color priority* analysis (gray line in lower right graph of Figure A2B) were submitted to a 2 × 6 repeated measures analysis of variance, with color appearance and timing as variables. There was no main effect revealing an advantage for a given color across timings (*M* = −9.4 ms), *F*(1, 13) = 1.3, *p* = 0.3. The interaction between object appearance order and timing was not significant, *F*(5, 65) = 0.8, *p* = 0.5. Data for the *direction priority* analysis (black line in lower right graph of Figure A2B) were submitted to a 2 × 6 repeated measures analysis of variance, with direction appearance order and timing as variables. There was no main effect of direction appearance order (*M* = −14.0 ms), *F*(1, 13) = 1.1, *p* = 0.3. There was no significant interaction between object appearance order and timing, *F*(5, 65) = 1.7, *p* = 0.1.

In summary, for the simpler questions of the form “Which is (Direction)?” there was a main effect of direction consistency such that responses were faster when the first object appeared on the side named by the directional term.
